# Plasma N-Glycoproteomics in monozygotic twin pairs discordant for body mass index reveals an obesity signature related to inflammation and iron metabolism

**DOI:** 10.1186/s13062-025-00609-y

**Published:** 2025-03-19

**Authors:** Maheswary Muniandy, Sakari Joenväärä, Birgitta W. van der Kolk, Tiialotta Tohmola, Hanna Haltia, Sina Saari, Antti Hakkarainen, Jesper Lundbom, Juho Kuula, Per-Henrik Groop, Jaakko Kaprio, Sini Heinonen, Risto Renkonen, Kirsi H. Pietiläinen

**Affiliations:** 1 Obesity Research Unit, Research Program for Clinical and Molecular Metabolism, Faculty of Medicine, University of Helsinki, Helsinki, Finland; 2 Transplantation Laboratory, Faculty of Medicine, University of Helsinki, Helsinki, Finland; 3 HUS Diagnostic Center, Helsinki University Hospital, Helsinki, Finland; 4 Department of Radiology, HUS Diagnostic Center, Helsinki University Hospital, Helsinki, Finland; 5 Faculty of Medicine Doctoral Program in Clinical Research, University of Helsinki, Helsinki, Finland; 6 Institute for Clinical Diabetology, German Diabetes Center, Leibniz Center for Diabetes Research, Heinrich Heine University, Düsseldorf, Germany; 7 Folkhälsan Institute of Genetics, Folkhälsan Research Center, Helsinki, Finland; 8 Research Program for Clinical and Molecular Metabolism, Faculty of Medicine, University of Helsinki, Helsinki, Finland; 9 Abdominal Center, Nephrology, University of Helsinki and Helsinki University Hospital, Helsinki, Finland; 10 Department of Diabetes, Central Clinical School, Monash University, Melbourne, VIC Australia; 11 Institute for Molecular Medicine Finland, FIMM, University of Helsinki, Helsinki, Finland; 12 Healthy Weight Hub, Abdominal Center, Helsinki University Hospital and University of Helsinki, Helsinki, Finland

**Keywords:** Obesity, Plasma, N-glycosylation, Proteomics, Monozygotic twin pairs

## Abstract

**Background:**

N-glycosylation is a complex, post-translational modification which influences protein function and is sensitive to physiological changes. Obesity is associated with alterations in protein function; however, little is known about the glycoproteome in obesity beyond observations of association with types and structures of selected glycopeptides. Most often, due to technical challenges, glycan composition and structure information are missing. Here, we combined label-free data-independent proteomics and targeted quantitative glycoproteomics to study N-glycosylation of plasma proteins in obesity. Using a monozygotic twin study design, we controlled for genetic variation and focused only on the acquired effects of obesity.

**Methods:**

Using plasma samples of 48 monozygotic twin pairs discordant for BMI (intrapair difference > 2.5 kg/m^2^), we identified using mass spectrometry, differential protein and glycopeptide levels between heavier and leaner co-twins. We used a within-twin paired analysis model and considered *p* < 0.05 as significant.

**Results:**

We identified 48 protein and 33 N-glycosylation expression differences (*p* < 0.05) between co-twins. These differences occurred either both in the protein expression and glycoprotein (sometimes in opposing directions) or independently from each other. Haptoglobin protein was upregulated (Fold Change = 1.10, *p* = 0.001) in heavier co-twins along with seven upregulated glycan compositions at N-glycosylation site Asn241. The complement protein C3 was upregulated (Fold Change = 1.08, *p* = 0.014) along with one upregulated glycopeptide at Asn85. Additionally, many glycopeptides were upregulated despite non-significant differences in protein-backbone plasma levels.

**Conclusion:**

Differential protein expression related to cholesterol biosynthesis and acute phase signalling as well as N-glycosylation of proteins related to iron metabolism and inflammation can be linked to acquired obesity.

**Supplementary Information:**

The online version contains supplementary material available at 10.1186/s13062-025-00609-y.

## Introduction

The World Obesity Federation, in 2024, estimated that over 1 billion people live with obesity (BMI ≥ 30 kg/m^2^) [1]. Obesity results from a complex interplay of genetic, epigenetic, and environmental factors [2]. While it has high genetic heritability, it is also influenced by lifestyle, socio-economic factors including education, childhood psychosocial factors, and emotional well-being [3, 4]. Obesity is a major public health concern that is associated with metabolic conditions that include insulin resistance, type 2 diabetes, cardiovascular diseases, and certain types of cancer [5]. Prevention and, failing that, timely treatment of obesity is thus pivotal in addressing this growing epidemic. Improved characterization of molecular alterations in obesity, which may contribute to metabolic derangements, is much needed.

One such molecular alteration is the N-glycosylation of proteins, a complex post-translational modification occurring on approximately half of all plasma proteins [6]. N-glycosylation is one of the most diverse protein modifications since there are as many possible N-glycan structures as structural branching possibilities. During protein production, the multi-span membrane protein complex oligosaccharyltransferase transfers the N-glycan precursors to the N-consensus sequence in the protein as the protein is being translated. After this point, glycosylation patterns start to evolve as glycans are modified by tissue specific enzymes, whose activity change in different pathophysiological states. These N-glycosylation changes can affect protein functionality and activity.

Fucosylation and sialylation are two glycosylation patterns often studied in human diseases [7, 8]. Sialylation of glycans regulates structural stability, cell recognition and communication and is important for immune response [9–11]. Fucosylation of glycans helps maintain a steady state in cells and tissues and has an important role in apoptosis [8]. Interestingly, although N-glycosylation is not template-driven, some N-glycans are heritable [12–14].

The relationship between proteins and their glycosylation patterns can vary; sometimes the glycoprotein profiles match the protein levels, while at other times, they do not. When observing microheterogenity (more than one variety of glycan at a specific glycosite [15, 16]) of one N-glycosylation site to the protein expression of that particular protein, the basic hypothesis in homeostasis is that they are similar in abundance and variety of the N-glycans produced. When we detect uncoupling of the qualitative or quantitative aspects of the N-glycosylation compared to the protein expression, we know that expression or the activity of the enzymes synthesizing or degrading the glycans have changed. These changes can be specific to a pathophysiological state and affect protein functions and activities [17]. Hence, these N-glycosylation alterations can be used as biomarkers of pathophysiological processes beyond what can be observed in proteins alone.

Aberrant plasma protein glycosylation is associated with a wide range of diseases, including cardiometabolic [18] and immunological disorders [19], and various cancer types [20–22]. For instance, proinflammatory immunoglobulin G (IgG) glycome traits associate with higher BMI [23, 24]. In individuals with obesity, higher levels of glycans with antennary fucose, sialylation and low-branching structures were found in the plasma of individuals after diet-induced weight loss [25–27]. Meanwhile, weight-loss following bariatric surgery associates with a decrease in core-fucosylated glycans and an increase in sialylated glycans [28].

The traditional method for N-glycan profiling involves cleaving glycans from the peptide chain and then analyzing the free glycan structures to detect global glycan-alterations [29]. Unfortunately, this process leaves the protein of origin for each glycan unknown, with the glycosylation-site information lost as well. As the type of glycan and its specific location within a glycopeptide can drastically change the behavior and characteristics of the protein, glycan/glycosylation site information may provide further valuable insights. Using our own previously developed workflow for quantitative N-glycoproteomics, we can not only quantify N-glycopeptides but also derive their peptide sequence, glycan composition, and proposed glycan structure [19, 21, 22, 30].

Obesity-related changes, in the plasma proteome have been extensively studied, revealing significant alterations in protein expression and function [31–35]. However, very little is known about the glycoproteome in obesity beyond observations of association with types (e.g. fucosylation and sialylation of glycans) and structures (e.g. simple, hybrid, complex) of only selected glycopeptides [23, 24, 36]. In this study, we addressed a critical gap in the literature regarding the effects of acquired obesity on both the global proteome and global glycoproteome levels using methodology that can identify glycan compositions at the glycosylated sites. By studying 48 rare monozygotic (MZ) twin pairs discordant for BMI (within-pair difference in BMI, ≥ 2.5 kg/m^2^), we identify genetically identical individuals with differing body weight. This is a unique and ideal design to isolate environmental and lifestyle factors from genetic influences. Our findings point at environmental factors that differ between the co-twins. Additionally, we uniquely combine results from both the proteins and glycopeptides to characterize obesity.

## Materials and methods

### Participants and study design

We selected 48 monozygotic twin pairs discordant for BMI from population-based longitudinal studies (FinnTwin16 (*n* = 2839 pairs [37]), FinnTwin12 (*n* = 2578 pairs [38]), and Older Finnish Twin Cohort (*n* = 2932 pairs [39]). These twin pairs, with within-pair BMI difference (ΔBMI ≥ 2.5 kg/m^2^), span two age groups (27–42 years old [*n* = 38 pairs] and 57–69 years old [*n* = 10 pairs]), with a mean age of 37.8. The participants included 29 female pairs. Smokers were categorised as either current smokers or non-smokers.

The study, approved by the Ethics Committee of the Helsinki University Central Hospital (protocol number 270/13/01/2008), adhered to the Declaration of Helsinki principles. All participants provided written informed consent.

### Body composition, energy intake and expenditure

We measured weight, height, whole-body fat (assessed by dual-energy x-ray absorptiometric scans), abdominal subcutaneous and visceral fat (measured using magnetic resonance imaging, MRI), and liver fat (measured using magnetic resonance spectroscopy, MRS) in the study participants as described here [40]. Additionally, we estimated participants’ physical activity with the Baecke questionnaire [41].

### Blood laboratory examinations

We collected fasting blood samples for blood biochemistry measures (analyzed from fresh samples using standardized methods at the HUSLAB laboratories) and the glycoproteomics analyses, using stored plasma samples kept at -80 °C.

### Proteomics and N-glycoproteomics analysis

Detailed information can be found in Supplementary Methods 1, and Supplementary Fig. 1 summarizes the proteomics and N-glycoproteomics workflows. Briefly, albumin was removed from the plasma samples and remaining proteins digested with trypsin. Peptide fractions and N-glycopeptide were isolated with size exclusion chromatography and both fractions were analyzed separately with LC-MS techniques. Since proteins that are most abundant in the plasma are also glycosylated, we did not deplete these proteins, except for albumin which does not have glycosylation sites. For proteomics, peptides were quantified and identified by LC-UDMSE runs.

For glycoproteomics, N-glycopeptides were first quantified (using size-exclusion chromatography) with LC-MSE runs. 16,000 N-glycopeptide ions were quantified and targeted for fragmentation with LS-MS2 techniques. Where possible, these peptide sequences were elucidated and the N-glycosylation site, the glycan composition, and proposed glycan structures investigated. The glycan compositions are shown, with the following abbreviations of each monosaccharide as follows: H: hexose; N: hexosamine; S; sialic acid; F: fucose. Importantly, the glycan structures are only proposed based on the composition and spectrum; these structures were not experimentally validated.

### Statistical analysis

#### Participant characteristics

Clinical variables are expressed as mean ± standard deviation (SD) for normally distributed variables, and as median (interquartile range) for non-normal variables. Differences between the co-twins (the heavier versus leaner co-twins) were assessed using paired Wilcoxon signed-rank test with *p* < 0.05 considered statistically significant.

#### Differential protein and N-glycopeptide analyses

We performed differential protein level analysis to identify differences in protein levels between co-twins. Additionally, we separately identified glycopeptides with different expression levels between co-twins. We used a paired samples analysis model (package *limma* in R-Bioconductor) to identify the non-genetic and non-shared environmental factors associating with the heavier/leaner status of the co-twins. Because not all twins were concordant for smoking status, we adjusted for it. We considered *p* < 0.05 statistically significant.

We identified:

1. proteins that were differentially expressed although related glycopeptides were not differentially expressed,
2. glycopeptides that were differentially expressed although related proteins were not differentially expressed,
3. proteins and related glycopeptides that were both differentially expressed,
4. proteins and glycopeptides for which we did not measure or identify their associated counterparts.

#### Biological pathway analysis

We further enriched the differentially expressed proteins using Ingenuity pathway analysis (Ingenuity Systems, Redwood City, CA, USA).

#### Clinical measures association analysis

We investigated, using linear mixed models (package R-lme4 [42]) with adjustments for sex, age, smoking and twinship, if any of the differentially (within-twin pairs) expressed proteins and glycopeptides associated with clinical measures.

#### N-glycosylation structural features analysis

Because sialylation and fucosylation of glycoproteins is altered in many diseases [7, 8], in addition to individually measured glycopeptides, we derived summary measures using the identified glycopeptides to calculate the total amount of sialyation or fucosylation of glycopeptides for each of the identified proteins. We used ratios to indicate, for each person, for each protein, the abundance of sialylated glycopeptides compared to the total abundance of glycopeptides as well as the abundance of fucosylated glycopeptides compared to the total abundance of glycopeptides. We used linear mixed models (package R-lme4 [42]) with adjustments for sex, age, smoking, and twinship to check if the amount of sialylation or fucosylation in a protein associate with clinical measures. Additionally, we also checked if the heavier co-twins had, in total, higher or lower amounts of sialylated or fucosylated proteins across all proteins with identified glycopeptides.

## Results

### Characteristics of study participants

As designed, the twin pairs displayed substantial discordance in all measures of adiposity. The heavier co-twins of the twin pairs (median BMI 30.5 [27.6–34.8]) had consistently higher measures of adiposity (i.e., body weight, BMI, body fat, fat-free mass, subcutaneous and intra-abdominal fat, adipocyte volume), insulin resistance, triacylglycerol, CRP, liver fat, and lower HDL compared to their leaner co-twins (median BMI 24.6 [22.1–27.7]) (Table 1). No significant differences were detected in total cholesterol, low density lipoprotein (LDL) cholesterol, or physical activity levels between the heavier and leaner co-twins. 14 twin pairs were discordant for smoking status (9 leaner co-twins and 5 unrelated heavier co-twins were smokers) and seven twin pairs were concordant for smoking status.

**Table 1:** Participant characteristics of 48 monozygotic twin pairs discordant for BMI; mean age 37.8 years (SD ± 14.1); 29 pairs (60%) were female

	Leaner co-twin	Heavier co-twin	*p* value
Sex (F/M)	29/19	29/19	
Body weight (kg)	76.2 ± 15.8	94.0 ± 18.6	1.68E-09
BMI (kg/m^2^)	24.6 [22.1–27.7]	30.5 [27.6–34.8]	1.68E-09
Body fat (%)	32.3 ± 9.45	40.8 ± 7.26	2.17E-09
Body fat (kg)	22.9 [17.5–34.8]	34.4 [30.5–46.1]	3.36E-09
Fat-free mass (kg)	48.1 ± 9.62	51.8 ± 11.0	1.40E-06
Subcutaneous fat (cm^3^)^a^	3635 [2611–4911]	5882 [4521–8165]	5.65E-07
Intra-abdominal fat (cm^3^)^a^	598 [330–1357]	1530 [861–2279]	8.94E-07
Liver fat (%)^a^	0.6 [0.4–1.1]	2.4 [0.7–6.1]	4.62E-05
Adipocyte diameter (µm)	79.6 [71.9–90.3]	90.3 [84.3–101]	1.02E-06
Fasting glucose (mmol/L)	5.24 ± 0.472	5.41 ± 0.526	0.051
Fasting insulin (mU/L)	4.7 [3.4–6.5]	7.9 [5.2–12.0]	5.95E-06
HOMA-IR index	1.08 [0.719–1.52]	1.93 [1.18–2.77]	8.59E-07
Matsuda index	7.93 [5.30–10.5]	4.34 [3.60–7.38]	5.39E-06
Total cholesterol (mmol/L)	4.45 [4–5.03]	4.40 [3.98–5.20]	0.321
HDL cholesterol (mmol/L)	1.59 [1.26–1.90]	1.28 [1.12–1.62]	1.21E-05
LDL cholesterol (mmol/L)	2.60 [2.13–3.23	2.85 [2.40–3.40]	0.057
Triacylglycerol (mmol/L)	0.88 [0.66–1.08]	1.12 [0.92–1.27]	0.005
CRP (mg/L)	0.6 [0.4–1.1]	2.4 [0.7–6.1]	3.17E-04
Adiponectin (µg/ml)	3241 [2336–4488]	2604 [1656–3496]	3.45E-04
Total physical activity (Baecke)	8.41 ± 1.48	7.95 ± 1.49	0.077
Smoking status	16	12	

Data are reported as mean ± SD (normally distributed variables) or median (interquartile range for skewed variables). We used Wilcoxon signed rank tests to calculate the p values and considered *p* < 0.05 significant. BMI, body mass index; HDL, high-density lipoprotein; LDL, low-density lipoprotein; HOMA-IR, homeostatic model for the assessment of insulin resistance; CRP, C-reactive protein. ^a^Data based on *n* = 26 twin pairs for whom these measures were available

### Overview of proteomics and glycoproteomics results

**Fig. 1:**
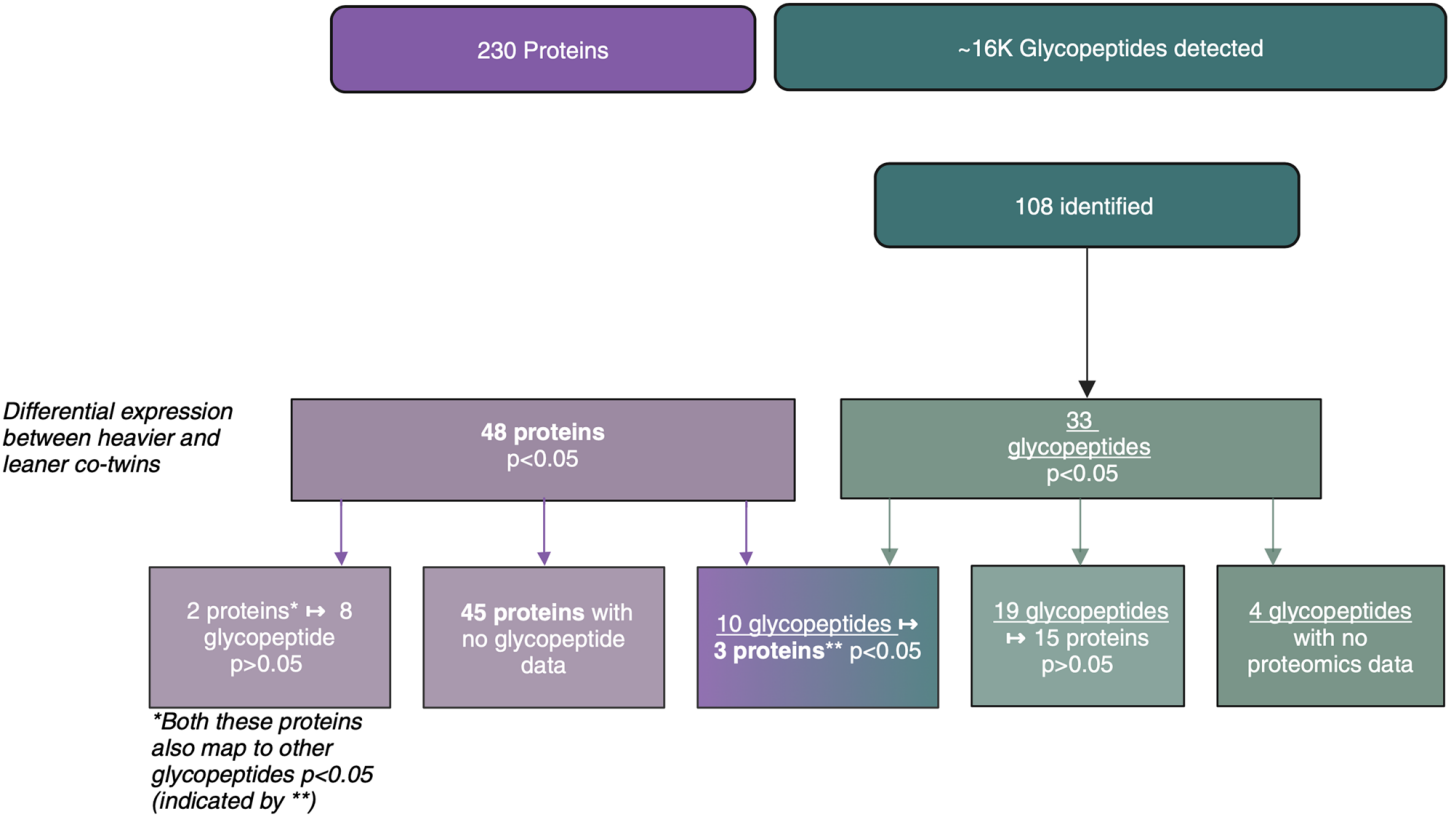
Overview of proteomics and glycoproteomics analyses results. Overall, 230 proteins were identified, with 48 proteins differentially (*p* < 0.05) expressed between heavier and leaner BMI-discordant co-twins. Out of these 48, three proteins were linked to ten glycopeptides which were also differentially (*p* < 0.05) expressed between co-twins. The remaining 45 proteins were either linked to glycopeptides not differentially expressed between co-twins (2 proteins) or not linked to any of the identified glycopeptides (43 proteins). Additionally, 108 glycopeptides were identified. Out of these 108, 33 glycopeptides were differentially (*p* < 0.05) expressed between co-twins. Out of these 33, only 29 has proteomics data as well. Ten of these 29 were linked to differentially expressed proteins. The remaining 23 glycopeptides were either linked to proteins not differentially expressed between co-twins (19 glycopeptides) or not linked to any of the identified proteins (4 glycopeptides). Additionally, we found 1986 differentially expressed glycopeptides that were detected but not traced to any of the proteins. (nominal *p* < 0.05 is considered significant)

Figure 1 provides an overview of both the proteomics and glycoproteomics results. Overall, out of the 230 identified proteins, 48 were differentially (*p* < 0.05) expressed between co-twins. Additionally, out of the 108 identified glycopeptides, 33 glycopeptides were differentially (*p* < 0.05) expressed between co-twins.

Most (35 out of 48) of the differentially expressed plasma proteins had higher expression in the heavier co-twins (Table 2, Supplementary Table 1). Several of these 48 proteins have been previously reported to be associated with obesity. For example, adiponectin (ADIPO) was one of the most significantly differentially expressed proteins with lower expression (FC = 0.85 *p* < 0.001, Table 2) in heavier co-twins.

**Table 2:** Top ten (by p-value) differentially expressed proteins between the heavier and leaner co-twins in the BMI-discordant pairs (*n* = 48 pairs/96 individuals). The paired co-twin analysis model was adjusted for smoking status

UniProt ID	UniProt Entry Name	Gene symbol	Protein name	Fold Change	*p* value	FDR *p* value
P01023	A2MG_HUMAN	A2M	Alpha-2-macroglobulin	0.91	3.60E-06	5.78E-04
Q15848	ADIPO_HUMAN	ADIPOQ	Adiponectin	0.85	5.78E-06	5.78E-04
Q9BRJ7	TIRR_HUMAN	NUDT16L1	Tudor-interacting repair regulator protein	1.17	7.54E-06	5.78E-04
P05452	TETN_HUMAN	CLEC3B	Tetranectin	1.15	9.23E-05	4.37E-03
O95568	MET18_HUMAN	METTL18	Histidine protein methyltransferase 1 homolog	1.25	1.06E-04	4.37E-03
P10721	KIT_HUMAN	KIT	Mast/stem cell growth factor receptor Kit	1.26	1.14E-04	4.37E-03
O15360	FANCA_HUMAN	FANCA	Fanconi anemia group A protein	0.89	2.82E-04	9.25E-03
A0A0U1RQE8	GLYLB_HUMAN	GLYATL1B	Putative glycine N-acyltransferase-like protein 1B	1.19	3.40E-04	9.78E-03
O95251	KAT7_HUMAN	KAT7	Histone acetyltransferase KAT7	1,14	4.22E-04	1.08E-02
Q9HCM2	PLXA4_HUMAN	PLXNA4	Plexin-A4	1,13	4.68E-04	1.08E-02

Similar to the protein expression patterns, most (31 out of 33) of the differentially expressed and identified glycopeptides were upregulated in the heavier co-twins; fold changes ranged from 0.84 to 1.40 (Table 3).

**Table 3:** Differentially expressed glycopeptides (with identified related proteins) between the leaner and heavier co-twins in the BMI-discordant pairs (*n* = 48 pairs/96 individuals). The paired co-twin analysis model was adjusted for smoking. Glycan composition abbreviations: H: hexoses, N: N-acetylhexosamines, S: sialic acids, F: fucose. Fold change indicates what happens in the heavier co-twins

Main protein function	Protein ID	Unitprot protein name	Glyco-peptide ID	*N* site	Glycan composition (WO Formylation)	Proposed glycan structure	Fold change	*P* value	FDR *p* value
Iron metabolism									
	P00738	HPT_HUMAN					1,103	0,001	0,018
			529	241	S1H5N4F2	Complex	1,379	2,10E-04	0,044
			29	241	S2H5N4	Complex	1,348	0,001	0,066
			131	241	S2H5N4	Complex	1,230	0,001	0,072
			1314	241	S1H4N4	Complex	1,403	0,002	0,106
			457	241	S3H6N5	Complex	1,195	0,002	0,110
			199	241	S1H5N4	Complex	1,238	0,006	0,184
			745	241	S1H6N3F2	Hybrid	1,249	0,008	0,202
	P00739	HPTR_HUMAN					0,942	0,035	0,184
			627	149,153	S2H5N4	Complex	1,145	0,001	0,077
			4828	126	H6N3F2	Hybrid	1,247	0,028	0,323
	P02790	HEMO_HUMAN					1,038	0,081	0,320
			809	187	S2H4N9F2	Complex	1,260	0,006	0,173
			350	453	S2H5N4	Complex	1,103	0,032	0,340
	P00450	CERU_HUMAN					1,010	0,696	0,833
			2445	138	S2H5N4F1	Complex	1,187	0,002	0,110
			1451	138	S1H7N4	Hybrid	1,151	0,003	0,120
			3892	138	S3H6N5	Complex	1,324	0,031	0,336
			3186	762	S2H5N4F1	Complex	1,391	0,032	0,341
	P02787	TRFE_HUMAN					0,999	0,954	0,971
			1486	432	S1H6N5	Complex	1,142	0,001	0,070
			195	432	H5N7	Complex	1,153	0,018	0,276
			1521	432	S1H5N3F1	Hybrid	1,160	0,019	0,282
Inflammation									
	P01024	CO3_HUMAN					1,084	0,014	0,089
			1337	85	H6N2	High Mannose	1,181	0,039	0,363
	P0C0L4	CO4A_HUMAN					1,013	0,695	0,833
			1000	1328	S2H5N4	Complex	1,113	0,025	0,310
	P01857	IGHG1_HUMAN					1,000	0,999	0,999
			1716	180	H3N10F1	Complex	1,189	0,006	0,184
	P01859	IGHG2_HUMAN					1,013	0,643	0,827
			2787	176	S1H11N11	Complex	1,298	0,003	0,120
			3777	176	H12N2	High Mannose	0,839	0,038	0,359
			4278	176	H4N3F1	Hybrid	0,908	0,040	0,366
			1751	176	S1H3N4F2	Complex	1,113	0,043	0,375
			3351	176	H3N3F2	Hybrid	1,218	0,048	0,388
	P01876	IGHA1_HUMAN					1,029	0,338	0,638
			5702	340	S2H5N5F1	Complex	1,302	0,004	0,141
	Q9GZX6	IL22_HUMAN					*not found*	*not found*	*not found*
			183	68	S1H4N3F1	Hybrid	1,181	0,003	0,120
Other									
	P02763	A1AG1_HUMAN					1,022	0,252	0,555
			1306	93	S2H7N6	Complex	1,137	0,019	0,281
			3182	56	H5N10	Complex	1,240	0,048	0,388
		CB071_HUMAN					*not found*	*not found*	*not found*
			5969	661	S2H5N4F2	Complex	1,120	0,026	0,314
	Q99572	P2RX7_HUMAN					*not found*	*not found*	*not found*
			1081	284	H6N4F1	Hybrid	1,276	0,003	0,120
	Q8TF74	WIPF2_HUMAN					*not found*	*not found*	*not found*
			3162	163	H6N11	Complex	1,317	0,016	0,267

### A third of the differentially expressed N-glycopeptides also mapped to differentially expressed proteins

Because some glycopeptide levels may also reflect protein levels, while other glycopeptides may be differentially expressed independently of the protein alterations, we sought to combine the results from the proteomics and glycoproteomics. Out of the 33 differentially expressed glycopeptides (Table 3, Supplementary Table 2), we found ten glycopeptides that mapped to three differentially expressed proteins: haptoglobin (HPT), haptoglobin-related protein (HPTR) and complement 3 (C3). Additionally, we found 19 differentially expressed glycopeptides mapping to eight proteins, mostly immunoglobulin subunits, which (in contrast to their glycopeptides) were not significantly different between the co-twins. The remaining four significant glycopeptides mapped to proteins that were not identified in our proteomics dataset (Table 3).

### On a protein level, acute phase signalling and cholesterol biosynthesis were upregulated

The top pathways enriched for these 48 differentially expressed proteins were related to acute phase signaling, DHCR24 signaling (related to cholesterol biosynthesis) and coagulation system (Supplementary Fig. 2), all three showed upregulation in the heavier co-twins.

### On a glycoproteome level, differential glycosylation patterns were observed in proteins involved in iron metabolism

**Fig. 2:**
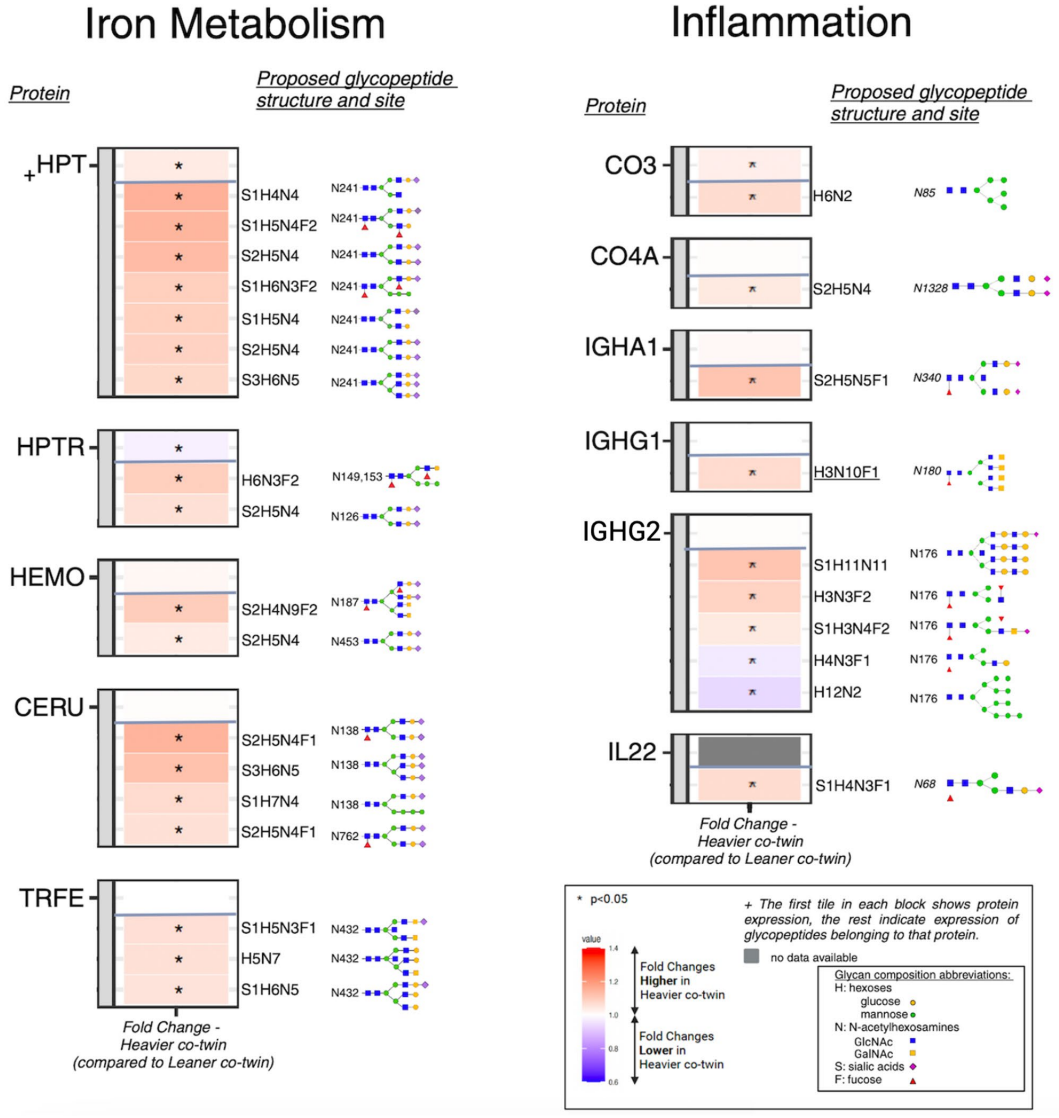
Heatmaps of the differential analysis results in relation to the iron metabolism and inflammation functions. The heatmap depicts fold changes between co-twins for the significant differentially expressed proteins/glycopeptides for the two main functions identified i.e. iron metabolism and inflammation. The colors of the heatmap indicate the strength of the fold changes i.e. red indicates higher values in heavier co-twins and blue lower values in heavier co-twins. *p* < 0.05 was considered significant, * *p* < 0.05

HPT, a hemoglobin scavenging protein in the plasma, showed significant protein upregulation (FC 1.10 *p* = 0.001) in heavier co-twins (Fig. 2; Table 3). Although this fold change is relatively small at the protein level, it may represent larger differences in absolute amount due to its nature of being a high-abundance plasma protein. Out of the four N-glycosylation sites for haptoglobin, we only found differential glycopeptides at site Asn241 indicating glycosylation microheterogeneity at this site. Out of the ten identified glycopeptides for HPT, we found seven different upregulated glycan compositions with fold changes 1.20–1.40. Interestingly, all these glycopeptides were sialylated with at least one sialic acid (Fig. 2; Table 3). One glycopeptide had a hybrid glycan structure, while the others had complex glycan structures. These results show increased sialylation at site Asn241 in the heavier co-twins. As all these glycopeptides have higher fold changes than the protein levels, it can be speculated that these glycosylation patterns are somewhat independent of the protein-level alterations.

HPTR, a protein with a similar function to haptoglobin, revealed three glycosylation sites and had a lower protein level (FC 0.94, *p* = 0.035) in the heavier co-twins. We identified eight glycopeptides for haptoglobin-related protein at two N-glycosylation sites (Asn126 and Asn149/153), out of which two glycan structures had significantly higher expression levels in the heavier co-twins (FC 1.15 and FC 1.25, *p* < 0.05) (Fig. 2; Table 3). In heavier co-twins, at site Asn126, one hybrid glycan structure had higher expression and at site Asn149/Asn153 one complex glycan structure had higher expression. Additionally, we also found eleven N-glycopeptides which were not different between the co-twins, that map to two differentially expressed proteins (HPT and HPTR) (Supplementary Table 2).

The protein levels of the heme-binding protein hemopexin, the iron-binding protein ceruloplasmin and the iron-oxidizing protein transferrin were not different between the co-twins (Fig. 2; Table 3). However, we identified altered glycopeptide abundances for all these proteins. For hemopexin, we found two out of five identified glycopeptides (with complex glycan structures at Asn187 and at Asn453) to be significantly higher in heavier co-twins. Moreover, we identified for ceruloplasmin four out of eight glycopeptides and transferrin three out of seven glycopeptides to be significantly higher in heavier co-twins (Fig. 2). Overall, we found upregulation in the glycopeptides related to iron metabolism; this pattern is not seen on the protein level.

### On a glycoproteome level, differential glycosylation patterns were observed in proteins involved in inflammation

Altered complement regulation in plasma is present in early stages of clinically healthy individuals with obesity [32]. In this study, the most abundant complement component, the glycoprotein complement 3, was upregulated (FC = 1.08, *p* = 0.014) in heavier co-twins. Complement 3 contains two N-glycosylation sites; we found one high-mannose glycopeptide at Asn85 to be higher in heavier co-twins (FC = 1.18 *p* = 0.039, Fig. 2; Table 3). Both the protein and the glycopeptide levels have similar fold changes, suggesting that the difference in the glycopeptide may reflect the difference in the protein level. We also found one disialylated, complex glycopeptide for complement 4a to be higher in the heavier co-twin (FC = 1.113 *p* = 0.025, Fig. 2; Table 3) with no significant differences in the related protein levels.

The IgG class of antibodies is composed of four different subtypes of IgG molecules, which can all be glycosylated at one glycosylation site. For IgG2, we identified 13 glycovariants of which five were differentially expressed at glycosylation site Asn176. Of those, two core-fucosylated glycopeptides with one attached fucose were downregulated in heavier co-twins (FC = 0.91 and 0.78 respectively) (Fig. 2; Table 3). The highest upregulated glycopeptide was a large monosialylated complex glycan with the composition S1H11N11 (FC = 1.30 *p* = 0.003), indicating potential terminal galactosylation and sialylation. Both can have a large impact on the IgG functionality; however a more detailed study of the actual structure of this composition [43] is needed. For IgG1, we identified 16 glycopeptides of which one (with a complex structure) was significantly upregulated in heavier co-twins. Of note, none of the IgG subclass protein levels were different between the co-twins, suggesting that the differences in these glycopeptides are unrelated to IgG-protein levels. Overall, we found upregulation in the glycopeptides related to inflammation; this pattern is not obvious on the protein level.

### Two-thirds of differentially expressed proteins associated with both adiposity and insulin resistance measures

**Fig. 3:**
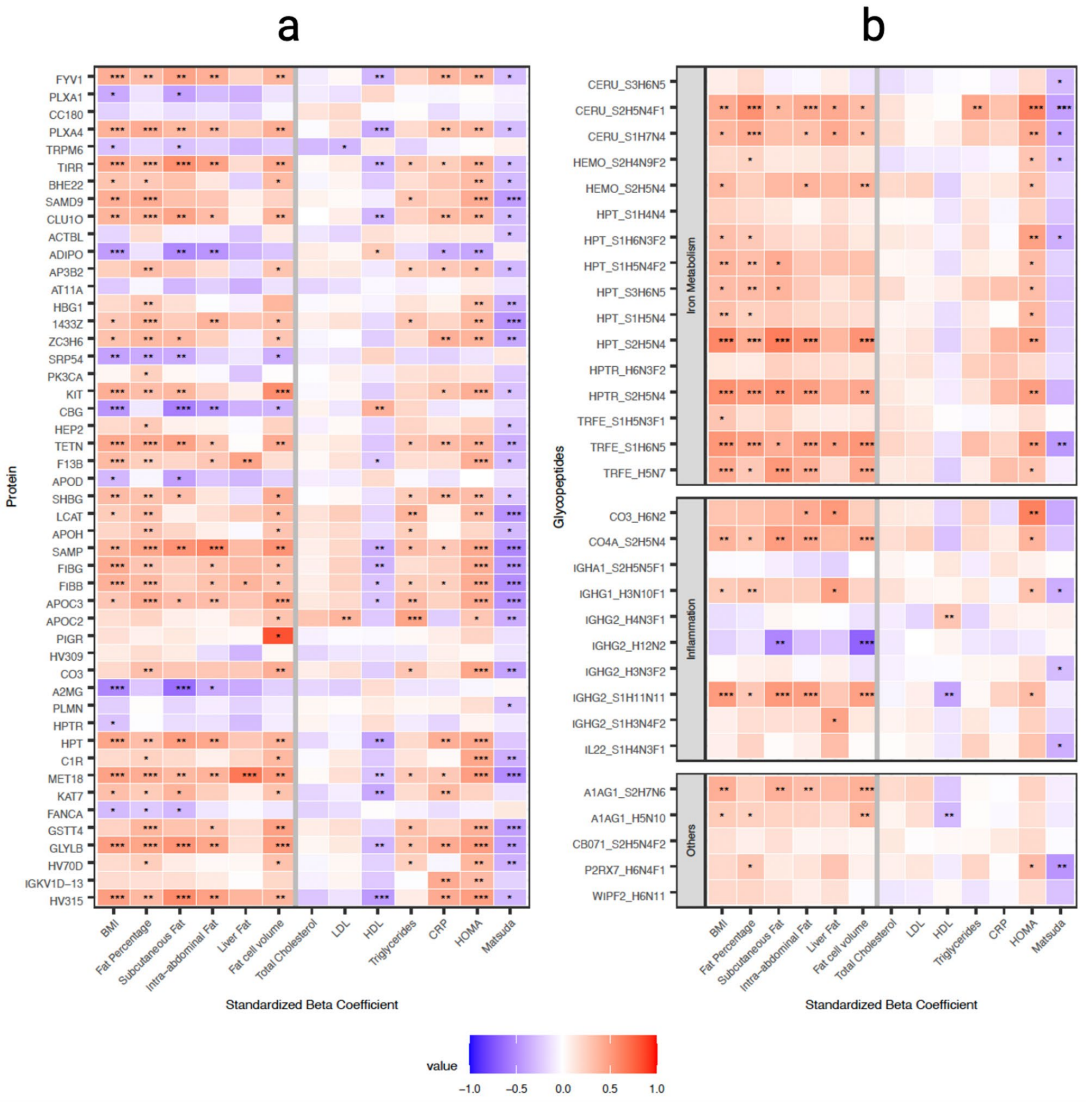
Heatmaps of the associations of differentially expressed proteins and glycopeptides to clinical outcomes. Standardized coefficients (β) showing associations (in standard deviations) between the protein levels and clinical measurements (figure a on the left) and associations (in standard deviations) between the glycopeptode levels and clinical measurements (figure b on the right). The colors of the heatmap indicate the strength of the association i.e. red indicates a positive and blue a negative association. FDR *p* < 0.05 was considered significant, * FDR *p* < 0.05, ** FDR *p* < 0.01, *** FDR *p* < 0.001. The glycopeptides are labelled with the protein name followed by the glycan structure with an underscore (i.e. “_”) between them

Given that we were able to identify molecular level differences in the proteome and glycoproteome in obesity, we next asked how these molecular profiles associated with clinical measures. Across all significant within-twin pair differentially expressed proteins, there were associations (Supplementary Table 3, Fig. 3) with several adiposity measures (BMI, fat percentage, subcutaneous and intra-abdominal adipose tissue amount, liver fat percentage, and fat cell volume), HDL, CRP, and insulin measures (HOMA index and MATSUDA index). Proteins APOC3, APOCH, KIT that were associated with at least one adiposity measure and one insulin measure are responsible for regulation of lipase activity. Proteins APOC2, APOC3, APOH, KIT, LCAT that associated with insulin measures are related to a host of lipid metabolism processes (protein-lipid complex organization, regulation of plasma lipoprotein particle levels, neutral lipid metabolic process, lipid homeostasis, glycerolipid metabolic process, lipid localization).

Protein HPT positively associated with most of the adiposity measures as well as HOMA with four of the HPT glycopeptides also showing positive associations with HOMA. Glycopeptides related to IgG and complement proteins also showed associations with adiposity measures and HOMA; CO3 showed a significant association to HOMA on a protein level as well.

### Glycopeptides that associate with both measures of adiposity and insulin resistance were sialylated

Across all significant within-twin pair differentially expressed glycopeptides, all glycopeptides that showed associations with at least one adiposity measures (BMI, fat percentage, subcutaneous and intra-abdominal adipose tissue amount, liver fat percentage, and fat cell volume), and at least one insulin measure (HOMA index and MATSUDA index) were also sialylated (Supplementary Table 4, Fig. 3). Most of these glycopeptides are related to iron metabolism proteins.

### Total amount of fucosylated or sialylated N-glycopeptides were different between co-twins

Bearing in mind that sialylation and fucosylation of glycans have been associated with several diseases and may also play a role in obesity, we, next, analyzed whether overall compositional features of the glycans, such as glycans being sialylated or fucosylated, were different between the co-twins and whether these features associated with clinical parameters. Across all the glycopeptides that were significantly different between co-twins and having protein level information, we found that heavier co-twins had significantly lower amounts of fucosylated N-glycopeptides (12.9% vs. 14.3%; *p* = 0.001). Conversely, they had significantly higher percentages of glycopeptides that were sialylated (66.5% vs. 65.3%; *p* = 0.027) (Supplementary Table 5).

On an individual protein level, heavier co-twins had a higher percentage of sialylated glycans attached to ceruloplasmin (*p* = 0.025) and a lower percentage of sialylated glycans on both transferrin (*p* = 0.031) and IGHG1 (*p* = 0.02073) compared to their leaner co-twins (Supplementary Table 6).

Additionally, we studied if the percentage of sialylation or fucosylation for a protein associated with clinical measures. The composition of glycans attached to transferrin showed that a lower amount of sialic acid was associated with higher adiposity, e.g. amount of subcutaneous fat (Supplementary Table 7, Supplementary Fig. 3) and CRP. HPT also associated negatively with adiposity and CERU associated positively with fat percentage and negatively with MATSUDA.

## Discussion

In this study, given the understanding that glycosylation affects protein function, we used a unique study design of MZ twin pairs discordant for BMI to profile the effects of excess body weight on the plasma proteome and glycoproteome. We confirm previous findings that acquired obesity, i.e. independent of genetic predisposition, is associated with increased levels of proteins related to cholesterol biosynthesis and acute phase signaling. Additionally, we reveal a glycopeptide profile related to iron metabolism and inflammatory proteins that associates with acquired obesity, findings that were not evident on a proteome level. We also show that most of the sialylated glycopeptides associate positively with measures of adiposity and insulin resistance. Importantly, we elucidate the glycan structures and sites and combine both proteomics and glycoproteomics results to obtain an enhanced view of acquired obesity. Our study not only reaffirms previous proteomics findings but also unveils new associations with plasma protein N-glycosylation that gives us more insights into acquired obesity not seen on a proteome level.

Glycoprotein profiles are diverse and dependent on protein expression along with the expression of glycan structures on the proteins. On the proteome level, we found upregulation of cholesterol biosynthesis and acute phase signaling, findings already linked to obesity [34]. On the glycopeptide level, we found iron metabolism and inflammation to be upregulated in the heavier co-twins. Not surprisingly, only a third of our differentially expressed glycopeptides also showed associations on proteomics levels. These obesity signatures that are missing from proteomics alone highlight the importance of glycoproteomics in characterizing obesity. For example, the significantly different iron metabolism glycopeptides that were upregulated in obesity only showed upregulation in two out of the five identified significantly differentially expressed proteins. Similarly, the differentially expressed inflammation-related glycopeptides that were upregulated in obesity only showed upregulation in one out of the six differentially expressed inflammation-related proteins.

Obesity has been linked to reduced absorption of iron and low serum iron levels [44, 45]. We found, in the heavier co-twins, higher levels of multiple glycopeptides that are related to iron metabolism [46]. Under normal conditions, iron is transported in the bloodstream by transferrin [46]. Ceruloplasmin oxidizes Fe^2+^ into Fe^3+^ to facilitate the binding of iron to transferrin [47, 48]. The scavenger proteins haptoglobin, haptoglobin-related protein and hemopexin protect tissues from oxidative damage by binding free hemoglobin and free heme, respectively [49]. In obesity, a condition of low-grade inflammation, the expression of hepcidin, a key regulator of iron homeostasis is stimulated [50]. Increased levels of hepcidin have been shown to inhibit the release of iron from macrophages thereby reducing intestinal iron absorption and decreasing serum iron levels [51]. Additionally, it is important to note that the proteins and related glycopeptides we discuss here, as part of our findings, are also acute phase proteins. Hence, the differences detected in the heavier versus leaner co-twins may point to both inflammation and related effects in iron metabolism protein functioning due to altered N-glycosylation.

In our study, we observed upregulation of several glycopeptides related to haptoglobin (HPT) and haptoglobin-related protein (HPTR). It is well known that N-glycosylation can affect protein folding [52, 53] and protein-protein interactions [54]. For instance, N-glycosylation can fine-tune the tight haptoglobin and hemoglobin interactions that are widely described as ‘irreversible’ by displaying microheterogeneity in the form of altered fucosylation and glycan branching at a specific N-glycosylation site [55]. Moreover, a molecular dynamic simulation study suggests that different glycans on the transferrin protein affect the layout of the iron binding site residue and the transferrin structure [56]. In our study, we show increased microheterogeneity for haptoglobin and transferrin. We also show that a lower percentage of sialic acids in the glycans attached to transferrin were associated with increased adiposity. It is tempting to speculate that these alterations may impact the protein function. Nevertheless, how the identified glycopeptide characteristics specifically attribute to iron metabolism in obesity remains to be studied.

Our findings also point to upregulation in glycopeptides related to complement 3 (C3) and complement 4 (C4). C3 is a central and the most abundant protein of the complement system, a key system for immune surveillance with a significant role in the pathogenesis of many diseases [57]. We have previously shown that deviant complement regulation in plasma is present in very early stages of clinically healthy individuals with obesity [32]. Here, we confirm our previous findings of complement regulation and observe an unprocessed, high-mannose (i.e. not complex nor hybrid) glycopeptide to be higher in heavier co-twins. Interestingly, in young people with type 1 diabetes, the C3 N-glycome has been reported to have more unprocessed glycan structures [58]. With regards to the C4A glycopeptide, we found significant positive associations with almost all the adiposity measures as well as HOMA making it a sensitive biomarker for both obesity and insulin resistance. Overall, however, it is important to note that the higher glycopeptide level may have been observed due to higher protein levels in the heavier co-twin.

It is also interesting to note that the highest upregulated IgG glycopeptide (composition S1H11N11) in heavier co-twins in our study is sialylated and associated with most of the adiposity measures as well as HOMA. IgG glycosylation has been well studied in cardiometabolic diseases [23, 24] and it has been shown that N-glycosylation determines the structure and immunological function of the IgG, including modulation of pro- and anti-inflammatory signaling and cellular immune response [59]. Inherent differences in IgG subclass specific glycosylation have been shown [60] and the different subclass glycosylation profiles found in our study, particularly in IgG2 compared to the other subclasses, could point towards their different biological role. For instance, the glycan on IgG is important as it interacts directly with the cell surface receptor, fragment crystallizable γ receptor (FcγR) backbone while also altering the orientation of the two CH2 domains, and potentially their flexibility, to affect FcγR binding [61]. It has also been shown that the addition of sialic acid converts IgG from proinflammatory into an anti-inflammatory agent [62]. In mice, reduced IgG sialylation has also been shown to be implicated in obesity-induced insulin resistance [63]. Due to the observational nature of our study, the etiological interpretation of our findings remains speculative.

Fucosylation and sialylation of glycans have been used as predictive biomarkers in many diseases for example inflammatory conditions and cancer [64, 65]. Serum sialic acid (glycoproteins with sialic acid attached to the oligosaccharide side chains) is a marker of acute-phase response [66] and has been shown to be higher in individuals with higher BMI and to associate with triglyceride levels [67]. Our results also show that the heavier co-twins have higher percentages of sialylated glycopeptides. One sialylated glycopeptide of CERU positively associated with triglyceride levels. Although there were no significant differences in HPT fucosylation between co-twins, we found that percentage of fucosylation in protein HPT associated positively with adiposity measures in our study. Higher fucosylation, a hallmark of M1 inflammatory macrophages [68], in our dataset may be hinting at a higher inflammatory profile in the twins with higher BMI in our dataset.

It is important to note that, as in the case of the proteome [69, 70], some N-glycosylation is heritable, with several glycans showing high heritability (> 50%) in plasma [71, 72]. This may explain the small fold changes that we observe for glycopeptides. In general, more abundant glycans tend to have higher heritability [72]. N-Glycans mostly attached to IgGs exhibit high heritability containing core-fucosylated biantennary N-glycans with low levels of sialylation. However, for several IgG glycans, essential contribution of environmental variance has been observed in the TwinsUK cohort [71]. Low heritability of acute-phase proteins N-glycans might reflect physiological changes connected with general metabolic health and/or inflammation [72] and within the twin pairs, this may be regulated at the level of epigenetics [71].

The strength of our study is many-fold. Firstly, out twin study model allows us to only focus on the acquired effects of obesity while controlling for genetics. We also overlay both proteomics and glycopeptide level data to understand the glycoproteome profile. Additionally, we retain the protein of origin for each glycan and the glycosylation-site specific information. Therefore, we can observe the site-specific behavior of individual N-glycosylation sites and consider microheterogeneity next to macroheterogeneity (i.e., unique combinations of glycans at different sites of the same protein) and metaheterogeneity (i.e., variation in glycosylation across multiple sites of a given protein) which is often observed in the traditional method for N-glycan profiling. Nevertheless, if glycan alterations are found at different glycosylation sites within the same protein, we are unable to determine whether these occur together within a protein, or separately. We also cannot determine the types of glycosidic bonds or tell apart monosaccharide epimers with our current analysis methods. Additionally, the functionalities of individual glycan/protein combinations are not well understood and require follow-up studies to understand their effects. Finally, we are not able to pinpoint which of the environmental factors (e.g. eating behaviors [73], socio-economic factors [74], physical activity [75], adverse childhood psychosocial factors [76], emotional wellbeing [77], medication [78]) associate with the differences in the co-twins.

## Conclusion

In conclusion, we show that the differential protein expression related to cholesterol biosynthesis and acute phase signalling as well as N-glycosylation of plasma proteins related to iron metabolism and inflammation can be linked to acquired obesity. We also show association on both proteins and glycopeptides to clinical measures. These distinct plasma glycoproteomics profiles in acquired obesity may provide directions for more personalized lifestyle or pharmacological interventions in the prevention of cardiometabolic disease. Notwithstanding, the underlying mechanisms that drive the observed differences require further study and future studies are urgently warranted to obtain detailed insight into these differential metabolic phenotypes.

## Electronic supplementary material

Below is the link to the electronic supplementary material.

**Supplementary Material 1:** Supplementary Methods

**Supplementary Material 2: Supplementary Fig. 1**. summarizes the proteomics and glycoproteomics workflows. For the proteomics experiment, peptides were quantified and identified by LC-UDMSE runs. For the glycoproteomics, the N-glycopeptides were first quantified with LC-MSE runs and statistically significant glycopeptides were later targeted for fragmentation with LS-MS2 techniques and peptide sequence, and glycan compositions identified

**Supplementary Material 3: Supplementary Fig. 2**. In total, 48 out of 230 proteins were differentially expressed between co-twins. The top 5 significant pathways from the Ingenuity pathway analysis (IPA) tool (*P* < 0.001) indicating which pathways are upregulated or downregulated in the heavier co-twins compared to the leaner co-twins. IPA z-scores for the pathways (calculated based on Fisher Exact test): upregulation (z-score > 0, red bar), downregulation (z-score < 0, blue bar), or no directionality (z-score = 0, grey bar). Here, all top 5 pathways were upregulated and so are all shaded in red with darker shades indicating increased upregulation. Results are ranked in order of statistical significance

**Supplementary Material 4: Supplementary Fig. 3**. Standardized coefficients (β) showing associations (in standard deviations) between the clinical measurements and percentage of protein sialylation or protein fucosylation. The colors of the heatmap indicate the strength of the association i.e. red indicates a positive and blue a negative association. FDR *p* < 0.05 was considered significant, * FDR *p* < 0.05, ** FDR *p* < 0.01, *** FDR *p* < 0.001

**Supplementary Material 5: Supplementary Table 1**. All within-twin pair differences in plasma proteins between the heavier and leaner cotwins in the BMI-discordant twin pairs. **Supplementary Table 2**. All within-twin pair differences in the glycopeptides in the BMI-discordant twin pairs (*n* = 48 pairs/96 individuals). **Supplementary Table 3**. Associations between protein expression and clinical measurements in the BMI-discordant twin pairs (*n* = 48 pairs/96 individuals). **Supplementary Table 4**. Associations between glycopeptide expression and clinical measurements in the BMI-discordant twin pairs (*n* = 48 pairs/96 individuals). **Supplementary Table 5**. Difference in percentage of fucosylation/sialylation across all proteins in the BMI-discordant twin pairs (*n* = 48 pairs/96 individuals). **Supplementary Table 6**. Difference in percentage of fucosylation/sialylation across each protein in the BMI-discordant twin pairs (*n* = 48 pairs/96 individuals). **Supplementary Table 7**. Associations between clinical measures to percentage of fucosylation/sialylation per protein in the BMI-discordant twin pairs (*n* = 48 pairs/96 individuals)

## Data Availability

Proteomics and N-glycopeptide data are uploaded to the PRIDE database [79]. The proteomics dataset has an accession PXD041584 and N-glycoproteomics PXD041384.

## Abbreviations

DIA: Data Independent Acquisition
LC-MS: Liquid Chromatography - Mass Spectrometry
UDMSE: Ultra Definition Mass Spectrometry
MSE: Elevated Energy Tandem Mass Spectrometry
CID: Collision Induced Dissociation
MRM: Multiple Reaction Monitoring
WE: Wideband Enhancement
