# Supplementary material for: Plasma N-Glycoproteomics in monozygotic twin pairs discordant for body mass index reveals an obesity signature related to inflammation and iron metabolism
Source: Biol Direct. 2025 Mar 19;20:31. doi: 10.1186/s13062-025-00609-y (PMC11921541; doi:10.1186/s13062-025-00609-y)
Supplement: Supplementary file 1 — Supplementary Material 1: Supplementary Methods [file 13062_2025_609_MOESM1_ESM.pdf]

Serum sample preparation for proteomics and glyco-proteomics

Desalting and albumin depletion

The samples were desalted using Zeba Spin desalting plates (96well 7k #89808; Thermo Fisher Scientific Ltd, MA, USA). Prior to sample loading, the buffer of the plate was changed to 25 mM Tris with 75 mM NaCl (pH~ 7-8) (Trizma® base, #T1503; Sigma-Aldrich, MO, USA) (Sodium chloride, #31434; Honeywell International Inc. NC, USA) according to the protocol to ensure maximal albumin binding in the following protocol. 70 µl of sample was pipetted to the plate and spinned through with centrifuging 1,000 g for two minutes in room temperature (Model 5810R; Eppendorf AG, Germany). Desalted samples were collected to new Eppendorf tubes (Protein LoBind® Tubes #0030108116, Eppendorf AG, Germany).

Albumin was depleted from the desalted samples with PierceTM Albumin Depletion Kit (#85160; Thermo Fisher Scientific Ltd, MA, USA). 400 µl of the resin slurry was used per sample and washed once with wash buffer according to the protocol. After this, 50 µl of the desalted sample was pipetted on top of the resin and allowed to incubate for two minutes. The unbound was centrifuged with 12,000 g for one minute in room temperature (Model 5415R; Eppendorf AG, Germany). The flow-through was pipetted again on top of the resin and incubated and centrifuged as previously. The resin was washed three times with 50 µl of the washing solution and collected to the same tube with previous flow-through.

Bradford assay

The protein concentration of the samples was determined with Bradford assay (PierceTM Coomassie Plus (Bradford) Assay Kit #23236; Thermo Fisher Scientific Ltd, MA, USA). A bovine serum albumin (BSA) standard was used in series of dilutions and the wavelength used for measuring was 595 nm (Sense microplate reader, model type 425-301; Hidex Oy, FIN). Total of 440 µg of protein was aliquoted and dried form each sample with a SpeedVac vacuum concentrator (Model DNA120; Savant Systems LLC, MA, USA). Dried samples were stored in -20°C until further use.

Trypsin digestion

Denaturation of sample proteins was performed with mass spectrometry suitable agent *Rapi*Gest SF Surfactant (#186002123; Waters Ltd Milford, MA, USA). The dried samples were resuspended with 0.2 % *Rapi*Gest SF solution in 50 mM ammonium bicarbonate (#09832; Sigma-Aldrich, MO, USA). The

samples were vortexed briefly and further denaturated in 99°C thermomixer (Thermomixer comfort; Eppendorf Ltd, DE) for 10 minutes with a hole in the lid of the sample tube.

Reduction of the denaturated sample proteins was carried out by adding dithiothreitol (DTT, #V315B; Promega corp. WI, USA) in the final concentration of 5 mM and incubating in 60°C water bath for 30 minutes. This was followed by alkylation with 15 mM final concentration of iodoacetamide (IAA, #57670; Sigma-Aldrich, MO, USA) incubated in room temperature and darkness for 30 minutes. Reduced and alkylated proteins were digested using sequence grade recombinant trypsin (Trypsin Gold, Mass Spectrometry Grade, #V5280; Promega corp. WI, USA). 33mM acetic acid was used to resuspend lyophilized trypsin and one µg of trypsin was added for every 100 µg of sample. The samples were incubated in 37°C and in darkness for 18 hours with the enzyme.

After the digestion, an acid treatment was carried out to remove *Rapi*Gest SF surfactant from the samples. A pH below two was reached with 0.5% end concentration of trifluoroacetic acid (TFA) (#302031; Sigma-Aldrich, MO, USA) to make the surfactant molecule insoluble. The samples were incubated in 37°C for 45 minutes and the surfactant molecules were then removed by centrifuging with 16,000 g in 8°C for 5 minutes (Model 5415R; Eppendorf Ltd, DE). The supernatant including the sample peptides was collected and dried with a SpeedVac vacuum concentrator (Model DNA120; Savant Systems LLC, MA, USA). Dried samples were stored in -20°C until further use.

Size exclusion chromotography

The dried samples were resuspended with 55 µl of 0.1% formic acid (FA, #5.33002; Sigma-Aldrich, MO, USA) and vortexed and sonicated until the pellet was resuspended. Remaining aggregates were pelleted down with a centrifugation of 10,000 g in 18°C for 10 minutes. 50 µl of the sample was injected to a size exclusion column to separate peptides and glycopeptides into different fractions (SuperdexTM 30 Increase 3.2/300 #29-2197-58; GE Healthcare, IL, USA). The column was run with a mobile phase of 0.1% FA at an isocratic flow rate of 0.08 ml/min and fractions including minutes 13-19 (glycopeptides, 560 µl) and 20-31 (peptides, 960 µl) were collected. The column was washed 30 minutes with the mobile phase between samples. 150 µl of peptide fraction and 135ul of glycopeptide fraction were dried with a SpeedVac vacuum concentrator (Model SPD121P; Thermo Fisher Scientific Ltd, MA, USA). The collected fractions and dried samples were stored in -70°C.

Peptide assay

Peptide assay was used to measure the peptide concentration of the dried peptide fraction (PierceTM Quantitative Fluorometric Peptide Assay #23290; Thermo Fisher Scientific Ltd, MA, USA). The dried fractions were resuspended by adding 50 µl of 0.1% FA and 2% acetonitrile in water (#5.33002; Sigma-

Aldrich, MO, USA) (ACN, #1.00029; Sigma-Aldrich, MO, USA) with vortexing and sonicating for 5 minutes. 10 µl of the sample was used for the peptide assay. The peptide samples were adjusted to a final concentration of 125 ng/µl and spiked in with internal Hi3 E.Coli standard (#186006012; Waters Ltd, Milford, MA, USA) in the final concentration of 50 fmol per injection in mass spectrometer. The dried glycopeptide samples were resuspended in 25 ul of 0,1% FA and 2% ACN in water without the internal standard for mass spectrometer.

Liquid Chromatography and Mass Spectrometry

The mass spectrometer used in of the following procedures was a Synapt G2-Si equipped with a NanoAcquity UPLC (ultra performance liquid chromatograph): both from Waters Ltd, MA, USA.
Synapt was calibrated with sodium iodide clusters in the positive mode with the mass range of 50-2500 m/z.

All the samples, peptides or glycopeptides, were trapped in a 75 µm x 250 mm C18 nanoACQUITY trapping column (#186006527; Waters Ltd, MA, USA), washed and eluted to the 75 µm x 250 mm C18 reverse phase nanoACQUITY analytical column (#186003815; Waters Ltd, MA, USA).
The gradient used is as follows: trapping with 2% B for 2 minutes and 8ul/min, then analytical gradient 0 minutes 2% B, 1 min. 8% B, 75 min 25% B, 78 min. 40% B, 80 min. 90% B, 87 min. 90% B, 90 min 2% B and 100 min 2% B with 450 nl/min. The gradient buffers used were 0,1% formic acid (#5.33002; Sigma-Aldrich, MO, USA) in water as buffer A and 0,1% formic acid in acetonitrile (#1.00029; Sigma-Aldrich, MO, USA) as buffer B.

A lock mass function for post calibration purposes was collected at 556.2771 m/z as a reference probe infusion at 1ng/ul in 50% acetonitrile in 1% formic acid water. Post calibration was done in proteomics and in N-glycopeptidomics.

Uniprot Homo sapiens reference proteome UP00000640 was used throughout for the protein sequence identification in this study (79038 protein sequences, gene count 20588 and Ensembl genome assembly GCA_000001405.27).

Proteomics

500ng of the peptide mixture was applied to the trap column and injected to the Synapt. The method used to collect the data was UDMSE (ultra definition mass spectrometry). The data was collected 50-2000 m/z, IMS (ion mobility mass spectrometry) wave velocity 650 m/s, scanning time one second,

the collision energy at low energy function was 20V and in the high energy function 60V. Raw data was collected and uploaded to the Progenesis QIP (Nonlinear Dynamics Ltd, MA, USA) for peptide/protein identification and quantification. The maximal FDR was 1%. Two unique peptides per protein were a minimum requirement for identification.

Quantification of the N-glycopeptides

N-glycopeptides are injected to the same ratio as their peptide counterparts. The analytical gradient is as follows: The data is collected in sensitivity MSE-mode (elevated energy tandem mass spectrometry), 100-2000 m/z, one second scan time, the collision energy at low energy function was 4V and in the high pPeptides were identified and removed from the analysis. The potential glycopeptide ions (m/z more than 500, charges three to five and abundances more than X) were sent to statistical analysis as described in detail below.

Fragmentation of the targeted N-glycopeptides

As the fragmentation of the N-glycopeptides is a targeted process, the N-glycopeptides were fragmented from the three samples that had highest abundance with three different fragmentation approaches by Collision induced dissociation (CID).

A) MRM

We used first MRM (Multiple reaction monitoring)-based method where target m/z and retention time was set in the parameters, but not the fragments. The data was acquired with one second scan time and 50-2500 m/z with a collision energy ramp in the trap 30 to 60V.

B) WIDEBAND ENHANCEMENT with ION MOBILITY

The pusher of the Synapt was synchronised to improve the intensities of either singly or doubly charged N-glycopeptide fragments. The targeted glycopeptides were fragmented twice in this method: firstly singly charged fragments and then doubly were enhanced. Peaks lists were combined.

Fragmentation data was acquired in resolution mode, 50-2500 m/z, one second scan time, retentions times were set, collision energy ramp was 30 to 60V in the trap and IMS mobility 600 m/s.

Peak list generation, glycopeptide ID and glycan structures

The peak lists were generated with MascotDistiller Version 2.7.1.0 (Matrix Science Inc, MA, USA)

The fragments and precursors were post calibrated with the lock mass 556.2771 m/z.

The peak lists were uploaded to the GlycopeptideID (Applied Numerics Ltd, Finland) web-based tool to identify the N-glycopeptides. Briefly, each spectrum was searched against the UniProt tryptic peptide database of known serum proteins allowing 2 missed cleavages and given a peptide score. Next, possible glycan compositions are fitted into the total mass, and the resulting glycopeptides are fitted into the spectrum to give a glycan score. The total score is the combination of the peptide score+glycan score. The results are ranked, and for each possible result an annotated spectrum is shown along with the b/y ions from the spectrum that match the most likely peptide chain. The glycan compositions are also shown, with the following abbreviations of each monosaccharide as follows: H: hexose; N: hexosamine; S; sialic acid; F: fucose. We used error margin of 18 ppm for the precursors and 5 ppm for the fragments, protonated ions, FDR for the amino acid sequence maximally 4%, 100 fragments per spectra included to the analysis, peptide+HexNAc fragment was required, methionine oxidation as a variable modification and cysteines were alkylated as fixed modifications. Importantly, the glycan structures are only proposed based on the composition and spectrum; these structures were not experimentally validated.
