# Supplementary figures and images for: Plasma N-Glycoproteomics in monozygotic twin pairs discordant for body mass index reveals an obesity signature related to inflammation and iron metabolism
Source: Biol Direct. 2025 Mar 19;20:31. doi: 10.1186/s13062-025-00609-y (PMC11921541; doi:10.1186/s13062-025-00609-y)

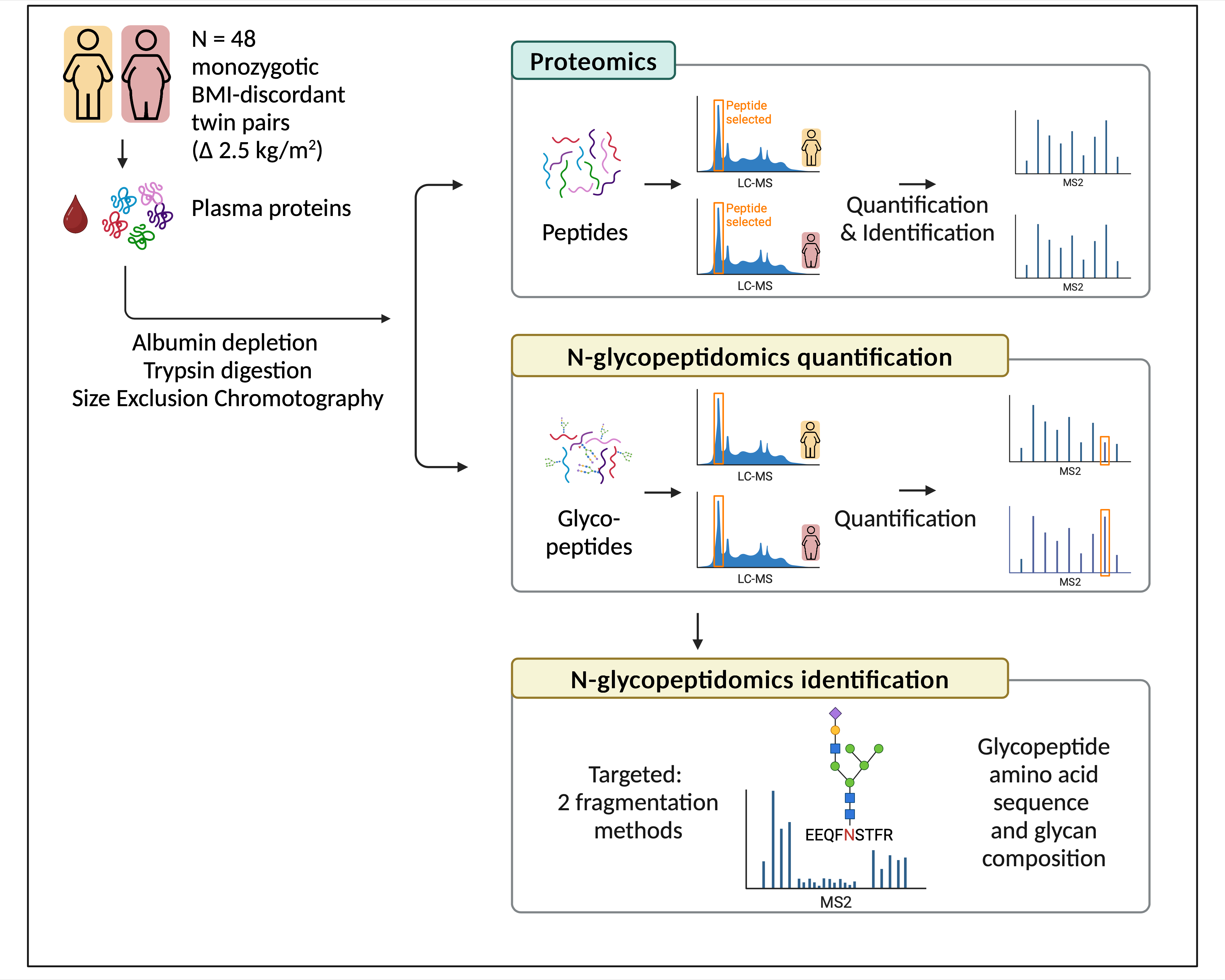

Supplement: Supplementary file 2 — Supplementary Material 2: Supplementary Fig. 1. summarizes the proteomics and glycoproteomics workflows. For the proteomics experiment, peptides were quantified and identified by LC-UDMSE runs. For the glycoproteomics, the N-glycopeptides were first quantified with LC-MSE runs and statistically significant glycopeptides were later targeted for fragmentation with LS-MS2 techniques and peptide sequence, and glycan compositions identified [file 13062_2025_609_MOESM2_ESM.tif]

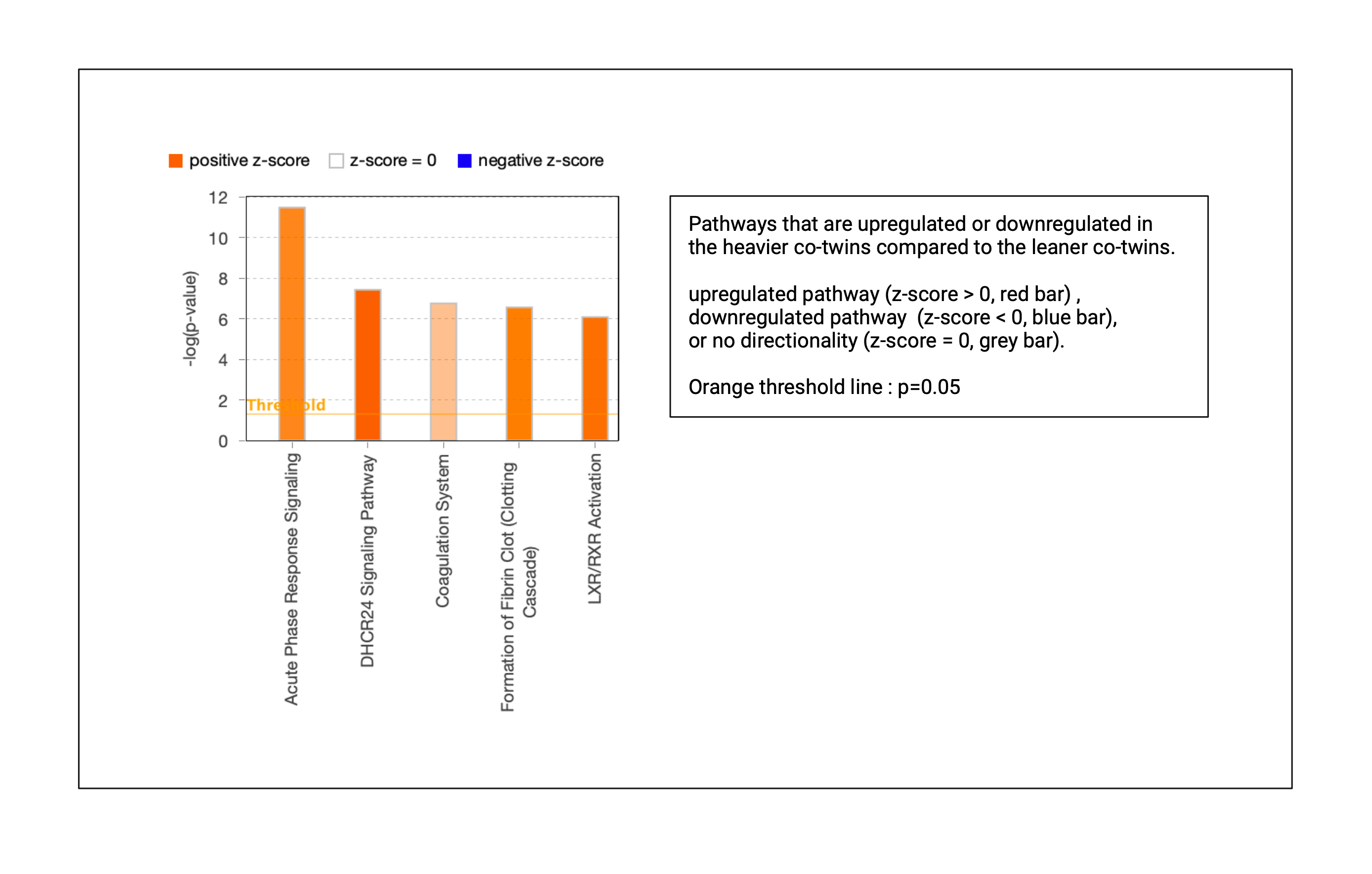

Supplement: Supplementary file 3 — Supplementary Material 3: Supplementary Fig. 2. In total, 48 out of 230 proteins were differentially expressed between co-twins. The top 5 significant pathways from the Ingenuity pathway analysis (IPA) tool (P < 0.001) indicating which pathways are upregulated or downregulated in the heavier co-twins compared to the leaner co-twins. IPA z-scores for the pathways (calculated based on Fisher Exact test): upregulation (z-score > 0, red bar), downregulation (z-score < 0, blue bar), or no directionality (z-score = 0, grey bar). Here, all top 5 pathways were upregulated and so are all shaded in red with darker shades indicating increased upregulation. Results are ranked in order of statistical significance [file 13062_2025_609_MOESM3_ESM.tif]

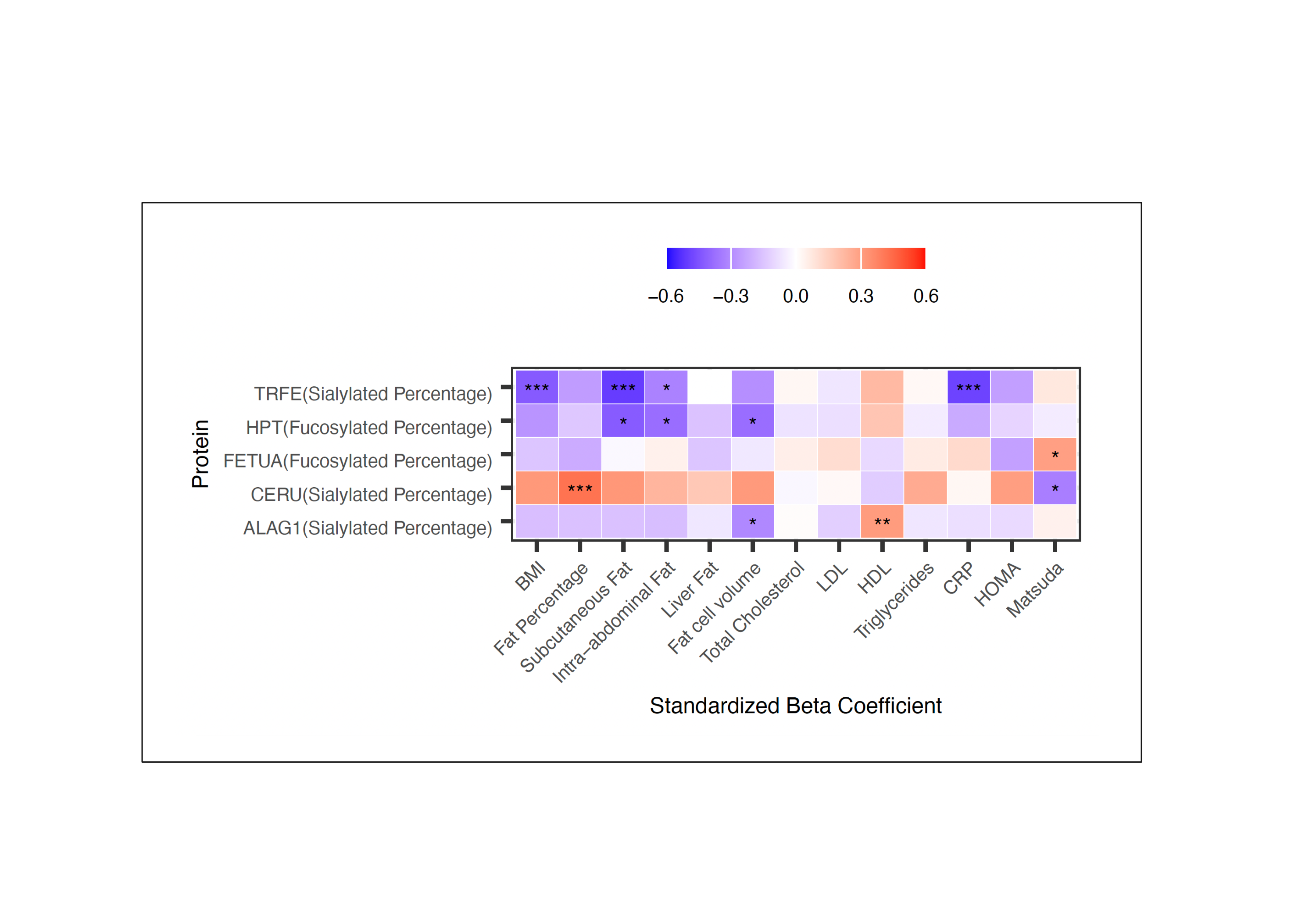

Supplement: Supplementary file 4 — Supplementary Material 4: Supplementary Fig. 3. Standardized coefficients (β) showing associations (in standard deviations) between the clinical measurements and percentage of protein sialylation or protein fucosylation. The colors of the heatmap indicate the strength of the association i.e. red indicates a positive and blue a negative association. FDR p < 0.05 was considered significant, * FDR p < 0.05, ** FDR p < 0.01, *** FDR p < 0.001 [file 13062_2025_609_MOESM4_ESM.tif]
